# Purification, structural characterization and immunological activity of *Sibiraea laexigata (L.) Maxim* polysaccharide

**DOI:** 10.3389/fnut.2022.1013020

**Published:** 2022-09-15

**Authors:** Xuhua Yang, Honghai Liu, Jutian Yang, Zhongren Ma, Penghui Guo, Hong Chen, Dandan Gao

**Affiliations:** ^1^China-Malaysia National Joint Laboratory, College of Life Sciences and Engineering, Northwest Minzu University, Lanzhou, China; ^2^Technology Research and Development Center, Gansu Tobacco Industry Co., Ltd., Lanzhou, China; ^3^Taizishan Ecosystem Observatory of Carbon Neutralization, Northwest Minzu University, Lanzhou, China

**Keywords:** *Sibiraea laexigata (L.) Maxim*, polysaccharides, purification, structural characterization, immunological activity

## Abstract

*Sibiraea laexigata (L.) Maxim* (SLM) has been used as an herbal tea for treating stomach discomfort and indigestion for a long time in china. Polysaccharides have been identified as one of the major bioactive compounds in the SLM. In the present paper, ultrasonic-assisted enzymatic extraction (UAEE) method was employed in polysaccharides extraction derived from SLM using polyethylene glycol (PEG) as extraction solvent, two SLM polysaccharides (SLMPs) fractions (SLMPs-1-1 and SLMPs-2-1) were purified by DEAE Cellulose-52 and Sephadex G-100 chromatography in sequence. Then, the preliminarily structure of the two factions were characterized by chemical composition analysis, molecular weight measurement, UVS, HPLC-PMP, FT-IR, nuclear magnetic resonance (NMR) spectra analysis and SEM. The results showed that SLMPs-1-1 and SLMPs-2-1 with different molecular weights of 1.03 and 1.02 kDa, mainly composed of glucose (46.76 and 46.79%), respectively. The results of structural characterization from FT-IR, ^1^H NMR, and SEM revealed that SLMPs-1-1 and SLMPs-2-1 contained the typical pyranoid polysaccharide with α-glycosidic bond and β-glycosidic bond. Furthermore, it was found that SLMPs-1-1 could increase the levels of tumor necrosis factor-α (TNF-α) and interleukin-2 (IL-2), and alleviated the immune organs tissue damage of cyclophosphamide (Cy)-treated mice. RT-qPCR and Western-Blot analysis showed that SLMPs-1-1 could significantly up-regulated the levels of NF-κB, TLR4, which revealed that SLMPs-1-1 could participate in immunosuppressive protection of Cy-treated mice. These findings suggested that the potential of SLMPs-1-1 as an alternative immunostimulator could be used in food and pharmaceutical industries.

## Introduction

Polysaccharides, a kind of natural macromolecular polymers, have been reported to have various biological activities, including antioxidant (1), anti-tumor (2) and enhancing immune activity (3), etc. In recent years, many natural plant polysaccharides have emerged as one of the hot topics and are widely perceived as ideal candidates for immunomodulatory agents in functional food (4) and practical medical fields due to their relatively low side effects and toxicity (5). For example, a polysaccharide derived from Astragalus has been developed as an immune enhancer using for adjuvant treatment of cancer in China (6).

*Sibiraea laexigata (L.) Maxim (SLM)*, which belongs to the *Rosaceae* family *(Genus Xianbei)*, mainly distributed in the shrub of 3,000–4,000 m in the western part of China. The aerial part of *SLM* is called “Liucha,” which has been used as a herbal tea and is typically utilized for the treatment of stomach discomfort and indigestion by Tibetans in Tibetan folk medicine of China for a long time (7). It has been found that the significant effective ingredients of SLM include polysaccharides (8), triterpenoids (9), flavonoids (10), and monoterpenes (11). However, previous studies about SLM mainly focused on extensive phytochemical investigations and the extraction of crude SLMPs, lacking a detailed study of the structures characterization and their immunomodulatory effects *in vivo* (12).

In present work, SLMPs was extracted by PEG-UAEE method and purified by DEAE Cellulose-52 and Sephadex G-100 chromatography in sequence. Then, the preliminary structure characterization of SLMPs-1-1 and SLMPs-2-1 were measured by chemical composition analysis, molecular weight measurement, UVS, PMP-HPLC, FT-IR, nuclear magnetic resonance (NMR) spectra analysis, and SEM. Furthermore, the immunoregulatory of SLMPs-1-1 and underlying mechanisms were thoroughly investigated by modulating CTX-induced immunocompromised mice *via* TLR4 and NF-kB receptor signaling pathways.

## Materials and methods

### Materials and reagents

SLM leaves were obtained from Hezuo City (102°54′E, 34°58′N, Gansu Province, China), dried and ground in a BJ-400 high disintegrator (Yongkang Boou Instrument Co., Ltd., Shanghai, China), sieved (80 mesh), and stored at 4°C until use. 1-phenyl-3-methyl-5-pyrazolone (PMP), monosaccharide standard products and TFA were purchased from Sigma-Aldrich Chemical Co., Ltd. (Louis, United States). DEAE Cellulose-52, Sephadex G-100, Cytokine (IL-2 and TNF-α) ELISA kits and Total ribonucleic acid (RNA) Purification Kits were purchased from Solarbio Biological Reagent Co., Ltd. (Beijing, China). Injectable cyclophosphamide (Cy) and Injectable levamisole (LH) were purchased from Shanghai Sangon Biotech Co., Ltd. (Shanghai, China). All other reagents used in experiments were all analytically pure.

### Extraction and purification of *Sibiraea laexigata (L.) Maxim* polysaccharides

The PEG-UAEE method was employed in crude polysaccharides extraction from SLM leaves (13). Briefly, 3.0 g pretreated SLM sample powder was immersed in a 45 mL aqueous PEG complex enzyme solution (E/S ratio of 21 U/g), the mixture was treated by a SB-500DTYultrasonic extraction equipment (Ningbo Xinzhi Biotechnology Co., Ltd., China) under an ultrasonic power of 400 W, ultrasonic times of 2.0 h, and ultrasonic temperature at 80°C. Then, the resultant extracts were centrifuged (Heraeus Multifuge X1R, Thermo Co., United States) at 5,000 r/min for 15 min, the supernatants were collected and concentrated to one-third of the initial volume using a RE52CS-1 vacuum distillation (Shanghai Yarong Biochemical Instrument Co., Ltd., China). The concentrated solution was sufficiently mixed with 3 times volumes of anhydrous ethanol and stored at 4°C for 24 h. The precipitate was dried by a LGJ-100F vacuum freezing dryer (Thermo Co., United States) at –80°C for 36 h to obtain the crude SLMPs (14). Sevage reagent was used to eliminate the proteins, and activated carbon was used to remove the pigment from the crude SLMPs (15). The yield of polysaccharides was calculated as:


(1) $$Y\left(\%\right)=\frac{W_{1}}{W_{0}}\times 100\%$$

Y is the yield of SLMPs (%, w/w), W_1_ is the weight of the crude SLMPs (g), and W_0_ is the weight of SLM leaves (g).

The crude SLMPs was re-dissolved in distilled water (20 mg/mL), then loaded onto a column of DEAE Cellulose-52 (50 cm × 2.6 cm) and successively eluted by the deionized water and NaCl solutions of different concentrations (0, 0.3, 0.6, 0.9, and 1.0 mol/L) at a flow rate of 1.0 mL/min (16). The absorbance of each tube was measured at 490 nm to analyze polysaccharide content of SLMPs according to the phenol–sulphuric acid method, then the eluents were dialyzed overnight at 4°C to remove salt, lyophilized, yielding two polysaccharide fractions (SLMPs-1 and SLMPs-2). These two purification fractions were further loaded on a Sephadex G-100 column (50 × 2.6 cm) eluting with deionized water (3 mL/min, 10 mL/tube) to afford SLMPs-1-1 and SLMPs-2-1, respectively.

### Structural characterization of *Sibiraea laexigata (L.) Maxim* polysaccharides-1-1 and *Sibiraea laexigata (L.) Maxim* polysaccharides-2-1

#### Chemical composition analysis

Phenol-sulfuric acid method was used to determine the total sugar content of SLMPs-1-1 and SLMPs-2-1 and glucose was used as a standard (17). The protein content of SLMPs-1-1 and SLMPs-2-1 were quantified by Bradford methods using bovine serum albumin (BSA) as a standard (18). The uronic acid content of SLMPs-1-1 and SLMPs-2-1 were estimated according to vitriol-carbazole method using D-glucuronic acid as the standard (19).

#### Molecular weight distribution

The molecular weights (Mw) of SLMPs-1-1 and SLMPs-2-1 were determined using a Waters 1260 Infinity HPLC system (Waters Co., United States) with 2410 differential refractive index detector and Ultrahydroge1TM-inear (300 × 7.8 mm, 8 μm, Agilent Co., United States) column. Dextrans (MWs: 1, 5, 10, 21, 40, and 84 kDa) were used as standards for calibration. The column temperature was maintained at 40°C, with 0.1 mol/L NaNO_2_ solution as the mobile phase and a flow rate of 1.0 mL/min (20). The regression equation of the standard curve was log_*Mw*_ = –0.6493x + 6.1564 (*R*^2^ = 0.9987); where Mw is the molecular weight; x is the retention time (min).

#### Monosaccharide composition analysis

The monosaccharide compositions of the purified SLMPs-1-1 and SLMPs-2-1 were determined according to Chen et al. with slight modification (21). 10.00 mg of freeze-dried SLMPs-1-1 and SLMPs-2-1 were treated with TFA (2 mol/L, 5 mL) at 110°C for 5 h. After cooling, the pH of the mixture was adjusted to 7.0 with NaOH (3 mol/L) and centrifuged to obtain supernatant. Then, 0.2 mL supernatant, 0.2 mL PMP methanol solution (0.5 mol/L), and 0.2 mL NaOH solution (0.3 mol/L) and were mixed and reacted at 70°C for 1 h. Finally, the reaction solution was neutralized by adding 1 mL trichloromethane and 0.1 mL HCl solution (0.5 mol/L), extracted with chloroform, repeated 3 times, centrifuged and collected the supernatant, which was filtered through 0.22 μm membrane and used for monosaccharide composition analysis by HPLC.

The determination process of monosaccharide composition was carried out with an Agilent 1260 HPLC system (ARC, Agilent Co., United States) equipped with a C_18_ column (4.6 × 250 mm, 5 μm, Agilent Co., United States) and a DAD detector. The mobile phase was a mixture of phosphate buffer (0.02 mol/L, pH 6.8) and acetonitrile in a ratio of 81: 19 (v/v) at flow rate 1.0 mL/min with column temperature of 28°C, and monitored at 250 nm. The monosaccharide standards, including Rha, Glu, Gal, Fru, and Ara were analyzed by HPLC in the same way as above.

#### FT-IR spectrometric analysis

The purified SLMPs-1-1 or SLMPs-2-1 were mixed with spectroscopic-grade KBr powder (Sigma Aldrich Co., United States), ground, and then pressed into 1 mm pellets for spectral measurement in the frequency range of 4000–400 cm^–1^ using a Nicolet 6700 FT-IR spectrometer (Thermo Co., United States) (22).

#### Nuclear magnetic resonance analysis

The NMR sample was prepared by mixing the 20 mg freeze-dried SLMPs-1-1 or SLMPs-2-1 with 0.5 mL of deuterated water (D_2_O), and NMR spectra of different fractions were obtained using a Bruker AVANCE III HD 400 spectrometer (Bruker Co., Germany) equipped with 5 mm double-tuned BBO probe and operating at 300 MHz for ^1^H. Each experiment was carried out at 80°C using a single-pulse experiment, an acquisition time of 1.66 s, a recycle delay of 5 s, and a spectral width of 10 kHz. The spectra were referenced at 0.0 ppm (23).

#### Scanning electron micrograph analysis

The morphological features of purified SLMPs-1-1 and SLMPs-2-1 were analyzed by a Zesis EVO18 field emission scanning electron microscope under 20.00 kV (Bruker Co., Germany). To render the power conductive, SLMPs-1-1 or SLMPs-2-1 was fixed on the sample stage with conductive adhesive for gold spraying and the appearance morphology was observed under different multiples (24).

#### Congo red staining assay

The Congo red staining assay was carried out to analyze the triple-helix arrangements of SLMPs-1-1 and SLMPs-2-1 according to the method reported by Huang et al. (25). Briefly, 1.5 mL Congo red solution (0.2 mmol/L), 1.0 mL sample solution of polysaccharides (2 mg/mL), and 3 mL NaOH solution with different concentrations (0, 0.2, 0.4, 0.6, 0.8, and 1.0 mol/L) were mixed thoroughly and reacted at 28°C for 1 h. Furthermore, the full-wavelength scan of the reaction solution in different concentrations of NaOH solution was performed by a UV-visible spectrophotometer (UV-1800, Shimadzu, Japan) at a wavelength of 200–800 nm, respectively, and the maximum absorption wavelength of the sample reaction was recorded.

### Immune activity analysis

#### Animals treatment and experimental design

A total of 60 Female Balb/c mice (specific-pathogen free grade,18–24 g, 5 weeks) were purchased from the Laboratory Animal Center of Lanzhou University and kept in room temperature at 24 ± 2°C and relative humidity of 60 ± 5% under an automatic 12 h light/12 h dark cycle. Six groups (10 mice in each group) were used in experiments: group 1 (normal saline control, PS): animals were treated with saline (1–30 d); group 2 (cyclophosphamide, CTX): animals were administrated with cyclophosphamide (1–10 d) (80 mg kg^–1^⋅bw) and saline (11–30 d); group 3 (levamisole hydrochloride, LH): animals were administrated with cyclophosphamide (1–10 d) and levamisole hydrochloride (11–30 d); group 4 (High-SLMPs-1-1 doses, SLMPs-H): animals were administrated with cyclophosphamide (1–10 d) and SLMPs (11–30 d, 800 mg kg^–1^⋅bw); group 5 (Mid-SLMPs-1-1 doses, SLMPs-M): animals were administrated with cyclophosphamide (1–10 d) and SLMPs (11–30 d, 400 mg kg^–1^⋅bw); group 6 (Low-SLMPs-1-1 doses, SLMPs-L): animals were administrated with cyclophosphamide (1–10 d) and SLMPs (11–30 d, 400 mg kg^–1^⋅bw); At the end of the treatment, mice were sacrificed within 24 h, and their spleen and thymus were dissected under sterile conditions (26).

#### Determination the cytokine content in serum

The whole blood samples of mice in each group was obtained by taking eyeball under sterile conditions and the serum was separated by centrifugation (8,000 rpm, 5 min) at 4°C for 10 min. The levels of IgG, IFN-γ, IL-4, and TNF-α in mice serum were determined using a ELISA kits (Solarbio Biological Reagent Co., Ltd., Beijing, China) by the manufacturer’s instructions. The color intensity was read using absorbance (*A*_450_) by a tunable microplate reader (Fisher FC, Thermo Co., United States), and the concentration of different cytokine were calculated according to a standard curve (27).

#### Histological observations of spleen and thymus

The spleen and thymus tissues were fixed in 10% PFA for at 37°C for 24 h, washed by flowing water for 24 h, dehydrated in a graded series of ethanol, soaked in xylene for 5 min, embedded in paraffin, and sectioned at 5 μm using a Leica RM2255 a microtome (Leica Biosystems Inc., Germany). Then the paraffin sections were stained with hematoxylin and eosin (HE) method, and observed under an Olympus Simon-01 microscope (Olympus Optical Co., Japan) (28).

#### Ribonucleic acid isolation, cDNA synthesis and RT-qPCR

RT-qPCR analysis was employed in detection of the mRNA expression of TLR4 and NF-κB in mice spleens extracted from each group (29). The total RNA of mice spleens in each group was extracted using a Trizol reagent Kit (TransGen Biotech Co., China). The concentration and purity of RNA were determined by ultraviolet spectrophotometry at 260 and 280 nm, aliquoted and stored at –80°C for future use. RNA was reverse-transcribed to cDNA using a PrimeScript™ RT reagent kit with cDNA Eraser (Takara Biotechnology Co., Ltd., Dalian, China) according to the manufacturer’s introduction. RT-qPCR was performed to quantify mRNA expression by a CFX96 Real-time PCR System (Bio-Rad, Hercules, United States) with SYBR Green Real-time Master Mix (Toyobo, Japan). The PCR program was: 95°C, 10 min; 95°C, 15 s; 60°C, 30 s, 40 cycles; melting curve analysis 65→95°C to detect the fluorescence signal every 0.5°C –cycle, and the reaction system was 2.0 μL cDNA, 1.5 μL 2.5 μM primers, 7.5 μL 2 × RT-qPCR Mix, 4 μL ddH_2_O, a total of 15 μL. The used primers are presented in Table 1.

**TABLE 1:** The primer sequence for RT-qPCR.

Gene	Product (bp)	Primer pair	Primer sequence (5′–3′)
GAPDH	133	Forward	CCTCGTCCCGTAGACAAAATG
		Reverse	TGAGGTCAATGAAGGGGTCGT
NF-κB	212	Forward	CGAGTCTCCATGCAGCTACG
		Reverse	TTTCGGGTAGGCACAGCAATA
TLR4	151	Forward	GGAACAAACAGCCTGAGACACTT
		Reverse	CAAGGGATAAGAACGCTGAGAA

#### Protein sample preparation and western blot

A protein extraction Kit (Solarbio, China) was used to isolated the total protein of the spleen tissue in each group mice, and a BCA protein quantitative Kit (Solarbio, China) was used to measure protein concentration. Then the protein (20 μg) was separated by 10% SDS-PAGE gel, transferred onto a 0.2 μm polyvinylidene difluoride (PVDF) membrane through a trans-blot Turbo transfer system (Bio-Rad, Hercules, United States) for 10 min at 25 V. The membrane was blocked in 0.02 mol/L PBS buffer (containing 5% skim milk powder (w/v) and 0.05% Tween-20, pH 7.5) at room temperature for 1 h, then incubated in a primary antibody solution at 4°C for 24 h. Thereafter, the membrane washed by TBST, incubated with secondary antibody at room temperature for 1 h. The protein bands were observed by using the ECL Western Blotting Analysis System (Bio-Rad, Hercules, United States) on an Image Quant LAS 4000 mini imager (GE, Life Science, United States) (30).

### Statistical analysis

All the experimental data were analyzed by SPSS statistical software version 19.0 (SPSS, Chicago, United States). The significant differences of each groups were determined by using a one-way analysis of variance (ANOVA) and Duncan’s test, taking *P* < 0.01 as extremely significant difference, and *P* < 0.05 as significant difference. Each experiment was performed in triplicate, and the data are demonstrated as mean ± standard deviation (SD).

## Results and discussion

### Extraction, isolation and purification of crude *Sibiraea laexigata (L.) Maxim* polysaccharides

The crude SLMPs were extracted by PEG-UAEE method, alcohol precipitation, deproteinization, and freeze-drying with a yield of 10.95 ± 0.13%, which was calculated using the weight of the dried SLM leaves. Since PEG can provide more -OH groups, it can enhance the solubility of polysaccharides in water and thus increase the yield of polysaccharides (1).

**FIGURE 1:**
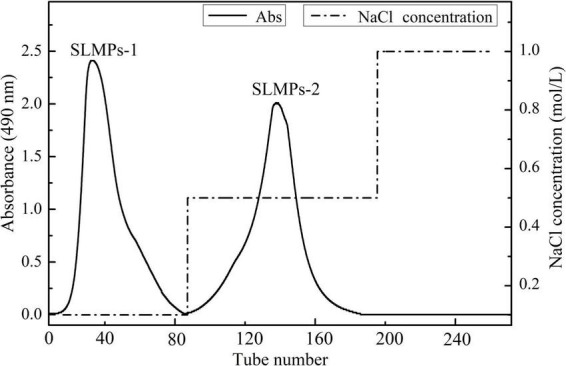
Elution profile of SLMPs by anion exchange chromatography on a DEAE-52 cellulose column.

**FIGURE 2:**
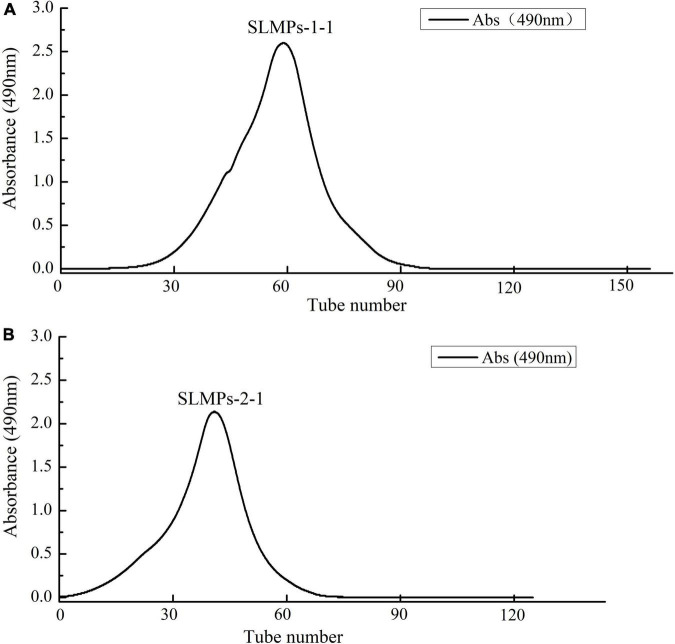
Elution curve of SLMPs-1-1 **(A)** and SLMPs-2-1 **(B)** on Sephadex G-100 column to obtain SLMPs-1 and SLMPs-2.

As shown in Figure 1, the crude SLMPs was firstly separated into two fractions (SLMPs-1 and SLMPs-2) purified by a DEAE cellulose-52 anion exchange chromatographic column on with gradient elution of 0–1.0 mol/L NaCl; These two fractions were collected, dialyzed, concentrated, freeze-dried, and loaded onto Sephadex G-100 gel filtration chromatographic column for further purification, respectively. As shown in Figure 2, each fraction generated only one single elution peak, representing SLMPs-1-1 and SLMPs-2-1, with yields of 61.4 and 54.9%, respectively. There is also a difference in the order of the collection tubes of SLMPs-1-1 and SLMPs-2-1, indicating that these two fractions are not only relatively pure single polymers, but also have different molecular weights.

### Characterization of the *Sibiraea laexigata (L.) Maxim* polysaccharides

#### Physicochemical property

As shown in Table 2, the total sugar contents of SLMPs-1-1 and SLMPs-2-1 were 82.08 and 81.64%, respectively, the two purified components had no significant difference. Table 2 also showed that the two fractions still contain a small amount of glycosyl-bound protein (0.18–0.21%), indicating that the protein was primarily removed by the Sevage method many times. However, after being separated and purified by DEAE cellulose-52 anion exchange chromatographic column and Sephadex G-100 gel filtration chromatographic column, the protein content of SLMPs-1-1 and SLMPs-2-1 were significantly reduced by 0.30 ± 0.94% and 0.33 ± 0.93% compared with SLMPs, indicating that the purified polysaccharide was relatively pure, and the process of purification were effective in removing the protein. Besides, the sulfate content in SLMPs-1-1 was significantly higher than that in SLMPs-2-1, and the two fractions were both acidic polysaccharides because they contained higher uronic acid content (22.4 and 20.3%, respectively). Many studies have shown that the polysaccharide can show good bioactive functions when sulfate content is higher, so SLMPs-1-1 will be preferred for further animal experiments (31).

**TABLE 2:** Chemical composition and relative molecular weight analysis of SLMPs-1-1 and SLMPs-2-1 ($\bar{x}$ ± s, *n* = 3).

Sample	Yield (%)	Total sugar content (%)	Protein content (%)	Sulfate content (%)	Uronic acid content (%)	Mw/Mn
SLMPs	10.95 ± 0.13	87.82 ± 0.17	0.52 ± 0.05	8.62 ± 0.12	21.8 ± 0.24	/
SLMPs-1-1	6.37 ± 0.15	82.08 ± 0.21	0.21 ± 0.11	6.51 ± 0.08	22.4 ± 0.17	1.03
SLMPs-2-1	4.58 ± 0.21	81.64 ± 0.18	0.18 ± 0.12	5.73 ± 0.09	20.3 ± 0.14	1.02

#### Ultraviolet spectral analysis

**FIGURE 3:**
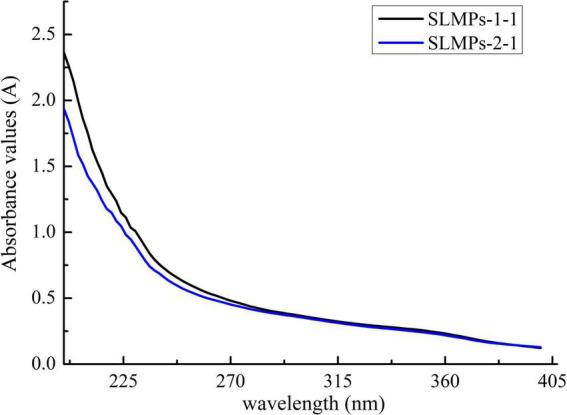
UV spectra of SLMPs-1-1 and SLMPs-2-1.

As shown in Figure 3, at a concentration of 1 mg/mL, SLMPs-1-1 and SLMPs-2-1 had no prominent absorption peaks appeared at the wavelengths of 260 and 280 nm in the UV spectral and negative responses to the Bradford test, which indicates absence of nucleic acid and protein in SLMPs-1-1 and SLMPs-2-1.

#### Molecular weight determination

HPGPC has been widely used in determination of polysaccharide molecular weight from different plants due to its advantage of rapid, great accuracy, good reproducibility, and high resolution (32). As shown in Table 2, both SLMPs-1-1 and SLMPs-2-1 are homogeneous polysaccharide, because they were gave a single symmetrical peak in Sephadex G-100 gel filtration chromatographic profile (Figure 2). The molecular weight of SLMPs-1-1 and SLMPs-2-1 were determined to be 1.29 × 10^3^ Da and 1.27 × 10^3^ Da on HPGPC in reference to standard glucans, respectively. Compared with the similar studies, the molecular weight of SLMPs was lower than other published polysaccharides (33). In addition, polydispersity values (Mw/Mn) could reflect the width of molecular mass distribution, thus the higher the polydispersity value represents the wider the distribution of polysaccharides. The value of Mw/Mn was close to 1, indicating that the two fractions had a relatively low polydispersity index and a homogeneity molecular weight distribution.

#### Monosaccharide composition analysis

**FIGURE 4:**
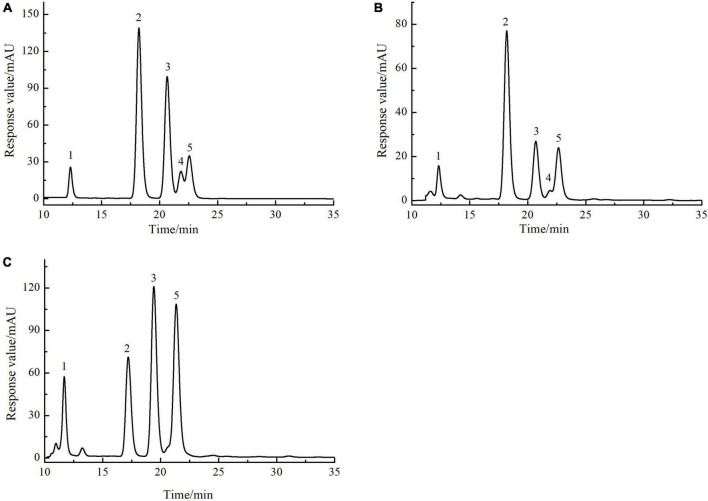
The HPLC chromatogram of monosaccharides of reference substances solution **(A)**, SLMPs-1-1 **(B)**, and SLMPs-2-1 **(C)**.

The monosaccharide composition of SLMPs-1-1 and SLMPs-2-1 were determined by HPLC-PMP (Figure 4). The results suggested that SLMPs-1-1 and SLMPs-2-1 had different monosaccharide compositions and content, although they were separated from the same native fraction SLMPs. SLMPs-1-1 and SLMPs-2-1 contained the same types of monosaccharides components (Ara, Glu, Gal, and Rha) and peak times. It may be since, during purification, the higher concentration of NaCl solution preferentially acts on the hydrogen bonds on the glycosidic bonds near fructose and arabinose, which reduces the degree of polysaccharide polymerization. SLMPs-1-1 was composed of Glu, Ara, Fru, Rha and Gal in the ratio (molar) of 46.79%: 13.96%: 13.04%: 8.69%:3.07%, and the monosaccharide composition of SLMPs-2-1 was Ara, Gal, Glu, and Rha, in molar ratio of 31.98%: 34.14%: 21.06%: 12.68%, indicating that the purification method might affect the structure and physicochemical properties of polysaccharides (34). Several studies have revealed that the biological activities of polysaccharides derived from different herbs primarily depend on these structural features, including inmonosaccharide composition and molecular weight (33).

#### FT-IR spectrum

**FIGURE 5:**
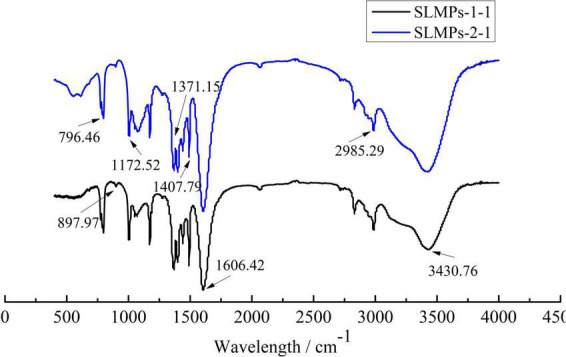
Infrared spectra of SLMPs-1-1 and SLMPs-2-1.

FT-IR spectra of SLMPs-1-1 and SLMPs-2-1 are compared in Figure 5. The robust ant broad absorption band at 3366.11 cm^–1^, which was attributed to the stretching vibration of O-H in the constituent sugar residues, and the strong absorbance band at around 2879.75 cm^–1^ was represented the stretching vibration of C-H in the sugar ring. These two absorbance peaks are characteristic of sugars, which proves that SLMPs-1-1 and SLMPs-2-1 were polysaccharide (35). The absorbance band at 1732.76 cm^–1^ was related to the bending vibration of bond water. Moreover, the absorption peak at 1436.25 and 1611.16 cm^–1^ were attributed to the carboxylic groups (COO-) and stretching vibrations of ester carbonyl groups (C = O), respectively, which indicated that purified SLMPs-1-1 and SLMPs-2-1 were acidic polysaccharides with uronic acid units, the locations of these peaks were similar to the investigations of *Atratylodes macrocephala* polysaccharides by FT-IR (36). This is consistent with the analytical results of monosaccharide compositions of SLMPs-1-1 and SLMPs-2-1. In addition, the absorbance band at 1,060 cm^–1^ was caused by C-O-C stretching and angular vibration in the sugar ring, the results suggested that the monosaccharide of purified SLMPs-1-1 and SLMPs-2-1 had pyranose rings. Moreover, the characteristic absorbance at 840.82 cm^–1^ suggested the presence of α-pyranose in SLMPs-1-1 and SLMPs-2-1, while the characteristic absorbance at 947.92 cm^–1^ related to the presence of a β-anomeric configuration in SLMPs-1-1 and SLMPs-2-1. It can be inferred that SLMPs-1-1 and SLMPs-2-1 were α- and β- type polysaccharides according to the more critical peak value of FT-IR spectra (37).

#### Nuclear magnetic resonance spectroscopic analysis

**FIGURE 6:**
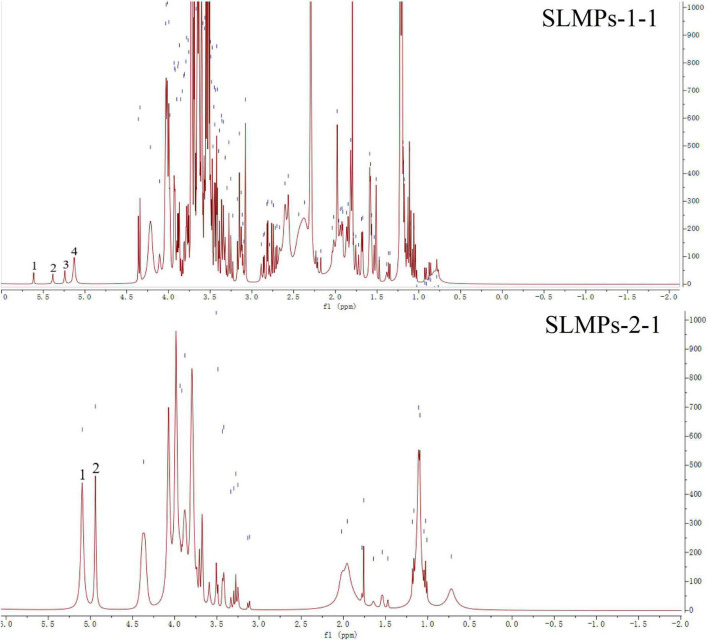
^1^H nuclear magnetic resonance (NMR) spectra of SLMPs-1-1 and SLMPs-2-1.

The structure identification and analysis of polysaccharides mostly use ^1^H NMR as the primary method to study the types of glycosidic bonds. The range of δ4.5–5.5 in the ^1^H NMR spectrum is the region where the proton signal mainly exists in the glycosidic bond of the polysaccharide, so there are several proton signals in this region in the ^1^H NMR spectrum, indicating that the sugar has several monosaccharide species. While other hydrogen signals are mainly concentrated in the narrow region of δ3.3–4.3, and the signal peaks overlap seriously. Among them, δ5.0 is the critical value of the proton signal to distinguish the configuration of pyranose. When the proton shift of the first carbon is more significant than 5.0, it is an α-glycoside, and when it is less than 5.0, it is a β-glycoside. The one-dimensional ^1^H NMR spectra of SLMPs-1-1 and SLMPs-2-1 can be seen in Figure 6. It can be seen from the figure that SLMPs-1-1 have 4 proton signal peaks, and in SLMPs-2-1, only two signal peaks were detected in the range of δ4.5∼5.5, this may be due to the overlap and interference between proton signals, leading to the lack of monosaccharide composition analysis (38), Furthermore, anomeric hydrogen appears in the range of δ4.2–4.4 and δ5.0–5.8 signal, indicating that there are both *α-*glycosidic bonds and β-glycosidic bonds in SLMPs-1-1 and SLMPs-2-1, which was consistent with the result from FT-IR analysis.

#### Microstructure analysis

**FIGURE 7:**
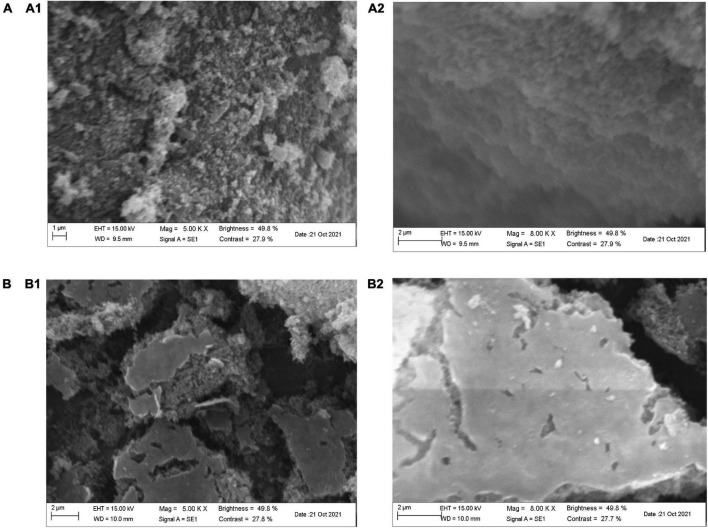
Scanning electron microscopy of SLMPs-1-1 and SLMPs-2-1. [**(A)** 5.00 KX; **(B)** 8.00 KX].

The microscopic scanning electron micrographs (SEM) structure of purified SLMPs-1-1 and SLMPs-2-1 (Figure 7) revealed that the single particle had irregular shapes, rough surface with different dimensions, which are typical characteristics of amorphous powders. The irregular-shaped particle also accompanied by fold structure with holes which is similar to the *Macroalgae* polysaccharides prepared by hot water extraction (39). It can be proved that SLMPs-1-1 and SLMPs-2-1 have a prominent amorphous structure and relatively complete structural morphology.

#### Conformational analysis

**FIGURE 8:**
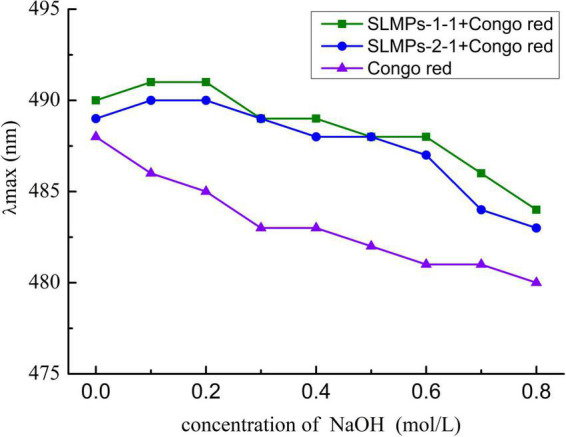
Conformation transition analysis of SLMPs-1-1 and SLMPs-2-1 at different concentration of NaOH.

The triple-helix arrangements of SLMPs-1-1 and SLMPs-2-1 were measured by Congo red test, and the results were displayed in Figure 8. It was apparently observed that the maximum absorbance wavelength of each sample in different concentration of NaOH (0.1–0.8 mol/L) had a certain degree of redshift compared with Congo red solution. The degree of redshift of SLMPs-1-1 was more dramatic than SLMPs-2-1, indicating that SLMPs-1-1 has a tighter triple-helical structure (40). With an increase in the concentration of NaOH, the degree of redshift of SLMPs-1-1, SLMPs-2-1 become smaller. This was because a high concentration of NaOH could destroy the hydrogen bond of the polysaccharide and induce the degradation of polysaccharides This indicated that SLMPs-1-1 and SLMPs-2-1 could form a regular ordered triple-helix structure in the neutral or weakly alkaline range (41).

### Effect of SLMPs-1-1 on serum IgG, IL-4, TNF-α, and IFN-γ levels

Immune globulin (Ig) and cytokines are mainly present in plasma, tissues and body fluids, playing an essential roles in immune response and regulation (42). As shown in Table 3, the serum levels of IgG, IL-4, TNF-α and IFN-γ in the mice treated with different SLMPs-1-1 concentrations were evaluated relative to CTX mice and PS group, respectively. It was found that IgG, IL-4, TNF-α and IFN-γ level in the CTX group was significantly lower than these in the PS group (*P* < 0.01), indicating that the CTX-treated mouse immunocompromised model was successfully established. IgG, IL-4, TNF-α, and IFN-γ level of each SLMPs-1-1 dose groups were significantly increased compared with the CTX group (*P* < 0.01), and the levels of IgG, IL-4, TNF-α, and IFN-γ in the mid-doses of SLMPs-1-1 was the highest in three different dose of SLMPs-1-1 groups (*P* < 0.05). Moreover, after 20 days of SLMPs-1-1 feeding, compared with the CTX group, the level of IgG, IL-4, TNF-α, and IFN-γ in the mid-dose of SLMPs-1-1 group was increased by 4.4, 8.13, 11.2, and 10.95%, respectively. This phenomenon indicated that SLMPs-1-1 could enhance immune regulatory through upregulating regulatory cytokines secretions (43).

**TABLE 3:** Comparison of plasma levels of IL-4, TNF-α, IFN-γ, and IgG in each group of mice.

Group	IL-4 (ng/mL)	TNF-α (ng/mL)	IFN-γ (pg/mL)	IgG (ng/mL)	*P*			
					
					IL-4	TNF-α	IFN-γ	IgG
PS	3.070 ± 0.09	2.722 ± 0.04	243.485 ± 2.22	3.812 ± 0.04				
CTX	2.831 ± 0.03	1.213 ± 0.05	165.983 ± 1.24	2.827 ± 0.05	0.000^➀^	0.000^➀^	0.000^➀^	0.000^➀^
LH	2.865 ± 0.03	2.570 ± 0.04	251.416 ± 0.84	3.576 ± 0.02	0.000^➁^	0.000^➁^	0.000^➁^	0.000^➁^
SLMPs-L	2.487 ± 0.04	2.115 ± 0.03	215.640 ± 0.69	3.267 ± 0.03	0.000^➂^	0.000^➂^	0.000^➂^	0.000^➂^
SLMPs-M	2.648 ± 0.02	2.307 ± 0.04	237.624 ± 1.31	3.412 ± 0.04	0.000^➃^ 0.004^➅^	0.000^➃^ 0.002 ^➅^	0.000^➃^ 0.000 ^➅^	0.000^➃^ 0.024 ^➅^
SLMPs-H	2.393 ± 0.03	1.704 ± 0.13	214.753 ± 1.27	3.174 ± 0.10	0.000^➄^ 0.007^➆^ 0.018^➇^	0.000^➄^ 0.003^➆^ 0.000^➇^	0.000^➄^ 0.008^➆^ 0.000^➇^	0.000^➄^ 0.004^➆^ 0.000^➇^

➀ Compare CTX group with PS group; ➁ Compare LH group with CTX group; ➂ Compare SLMPs-L group with CTX group; ➃ Compare SLMPs-M group with CTX group; SLMPs-L group comparison; ➆ SLMPs-H group compared with SLMPs-L group; ➇ SLMPs-H group compared with SLMPs-M group.

### Histological observations of spleen and thymus

**FIGURE 9:**
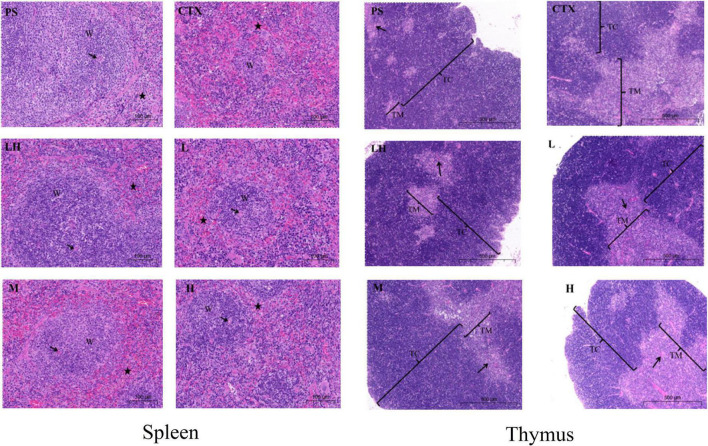
Effect of SLMPs-1-1 on spleen and thymus histomorphology in mice. → Represents periarterial lymphatic sheath, W represents white pulp, ⋆ represents red pulp.

As shown in Figure 9, the histopathology of the thymus and spleen were observed with an optical microscope. The histopathology of the spleen and thymus from PS group mice had clear medullar structure, visible the medulla inside and regular shape of the thymus cortex. Compared with that in the PS group, the boundary between the medulla and cortex was not clear, and the lymphatic sheath around the arteries was severely damaged in the CTX group, indicating that the model in this experiment was successful (44). Compared with the CTX group, the border between the red pulp and the white pulp were evident in the mid-dose group of SLMPs-1-1, and the boundary between the cortex and medulla was not evident in the high- and low- dose of SLMPs-1-1 groups, but slightly better than that in the CTX group. Similarly, in the LH group and mid-dose group of SLMPs-1-1, the increase in the area of white pulp was more prominent, and the white pulp and red pulp were close to the PS group. The results indicated that SLMPs-1-1 could prevent damage to the spleen and thymus cells in CTX-induced mice (45).

### Effects of SLMPs-1-1 on TLR4 and NF-κB mRNA expression in spleen

The relative gene expression levels of TLR4 and NF-κB in the spleen of CTX-induced immune deficiency model mice were detected by the RT-qPCR, aiming to demonstrate that SLMPs-1-1 can improve the immune suppression effect of mice by regulating the expression level of immune regulatory factors in the spleen.

**FIGURE 10:**
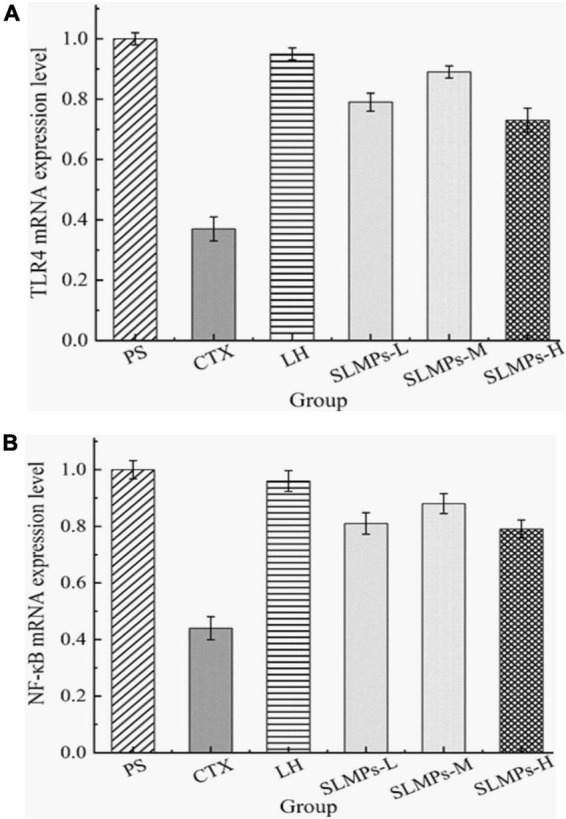
Effect of SLMPs-1-1 on the regulation of TLR4 and NF-κB gene expression in mouse tissues. **(A)** TLR4 gene expression in mouse tissues, **(B)** NF-κB gene expression in mouse tissues.

TLR4 is a membrane protein expressed on immune cells and epithelial cells (46), and its mainly distributed in macrophages, renal tubular epithelial cells, and other parts, which can be activated without the need for foreign pathogens to invade Innate immune inflammatory response (47). It mainly uses the MyD88-dependent pathway as a classic signal transduction pathway, and mediates the production of NF-κB to stimulate downstream inflammatory effects (48). Changes in the expression level of TLR4 mRNA detected by RT-qPCR in the spleen of different groups mice are shown in Figure 10A, a melting curve analysis has been used to verify the presence of a single gene-specific peak and the absence of primer dimer (49). The results showed the ratio of TLR4 mRNA in the CTX group and LH group was 0.44 and 0.96, and the mRNA expression level of TLR4 in SLMPs-1-1 first increased and then decreased with the increase of dose. The LH group and SLMPs-1-1 dose groups were significantly up-regulated (*P* < 0.01) compared with the CTX group, and there were significant differences between SLMPs-1-1 dose groups (*P* < 0.05), but the mid-dose group of SLMPs-1-1 had no significant up-regulation effect (*P* > 0.05). In conclusion, the effect of SLMPs-1-1 on the expression level of TLR4 mRNA in the spleen tissue of CTX-induced immunocompromised model mice was obvious, and the dose-response relationship was in the range of 200–400 mg/kg.

NF-κB is an essential to signal transduction, cell activation, and transcriptional activator in the TLR4 immune regulatory network’s downstream signaling pathway, which regulates cytokines levels, immune receptors, and anti-apoptotic proteins; therefore, NF-κB induces inflammatory immune responses (50). In addition, the dissolution curves of each hand have no signs of non-specific dissolution peaks or miscellaneous peaks, which confirmed the high specificity of the primers and indicated the amplified products have sure accuracy and can be used for experiments analyze (51). The mRNA expression levels of NF-κB in different dose groups were significantly decreased (*P* < 0.05, *P* < 0.01) compared to the PS group (Figure 10B). It was apparently observed that the mRNA expression levels of NF-κB in the LH group and each dose group were significantly increased (*P* < 0.01) compared with the CTX group. Among them, the mRNA expression level of NF-κB in the mid-dose group of SLMPs-1-1 was the highest (*P* < 0.01). This indicated that the dose of SLMPs-1-1 should be fully considered if the SLMPs-1-1 was used as an alternative immunostimulator in food and pharmaceutical industries.

### Effects of SLMPs-1-1 on related proteins expression in spleen

**FIGURE 11:**
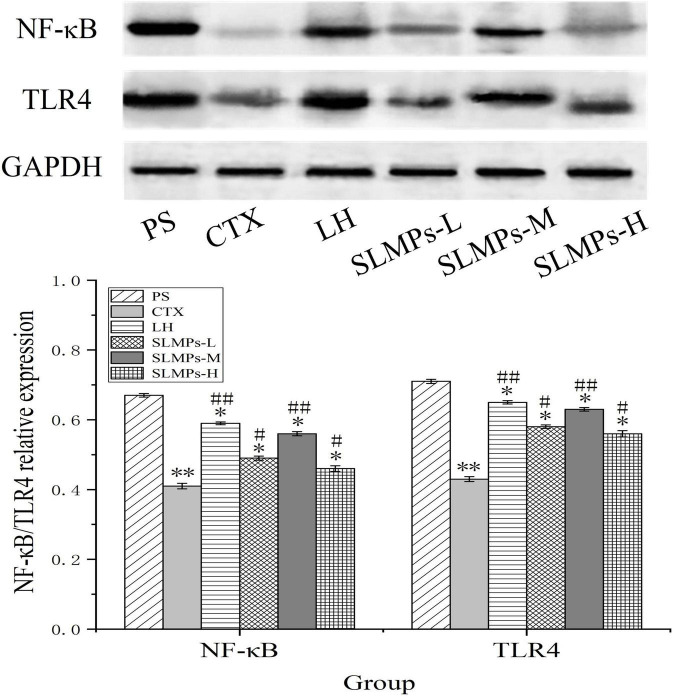
Effects of SLMPs-1-1 on the proteins expression of NF-κB and TLR4 in spleen [compared with PS group (*P < 0.05; **P < 0.01); compared with CTX group (^#^*P* < 0.05; ^##^*P* < 0.01)].

The protein expression levels of TLR4, and NF-κB in mice spleen were determined by western blotting (Figure 11). It was found that the proteins expression levels of NF-κB and TLR4 in the CTX group were significantly lower than those in the other groups (*P* < 0.05), and the proteins expression levels in the LH group and the mid-dose group of SLMPs-1-1 were higher than those in the CTX group (*P* < 0.05). The proteins expression levels of TLR4 and NF-κB were higher in the low- and high-dose group of SLMPs-1-1 group compared with the CTX group but were still significantly lower than those in the mid-dose group of SLMPs-1-1 (*P* < 0.05), and the dose of SLMPs-1-1 showed a dose-response relationship in the range of 200–400 mg/kg. This indicated that SLMPs-1-1 effectively improved the expression of NF-κB and TLR4 in the spleen tissue of CTX-induced immunocompromised mice, consequently playing a protective role during immune regulation.

## Conclusion

In this study, two SLMPs fractions (SLMPs-1-1 and SLMPs-2-1), with average molecular weight of 1.29 × 10^3^ and 1.27 × 10^3^ Da, respectively, were isolated and purified. The main components of the two polysaccharides were Glu and Gal (46.79 and 34.14%). The two polysaccharides fractions exhibited absorption peaks of characteristic α*-*glycosidic and β-glycosidic bonds pyranoid polysaccharides. The results showed that SLMPs-1-1 could accelerated recovery of spleen and thymus indexes, and up-regulate the levels of IgG, IL-4, TNF-α, and IFN-γ in the serum of the Cy-treated mice. SLMPs-1-1 also could improve the adaptive immune function by increasing the mRNA and protein expression of TLR4 and NF-κB. These results suggest that SLMPs-1-1 can be used as an immunostimulator to stimulate both the innate and adaptive immune responses for application in immunological diseases and functional foods.

## Data availability statement

The raw data supporting the conclusions of this article will be made available by the authors, without undue reservation.

## Ethics statement

The animal study was reviewed and approved by the Laboratory Animal Ethics of Northwest Minzu University.

## Author contributions

DG and ZM designed the whole experiment. XY and HC prepared SLMPs. JY, HL, and PG determined the structure of SLMPs. XY and DG wrote the manuscript. All authors discussed the results and contributed to the article.
